# The new temperature-sensitive mutation PA-F35S for developing recombinant avian live attenuated H5N1 influenza vaccine

**DOI:** 10.1186/1743-422X-9-97

**Published:** 2012-05-23

**Authors:** Wenting Zhang, Jiagang Tu, Zongzheng Zhao, Huanchun Chen, Meilin Jin

**Affiliations:** 1 Unit of Animal Infectious Diseases, National Key Laboratory of Agricultural Microbiology, College of Veterinary Medicine, Huazhong Agricultural University, Wuhan, 430070, P. R. China

**Keywords:** Influenza, H5N1, Vaccine, Live attenuated

## Abstract

**Background:**

H5N1 highly pathogenic avian influenza virus (HPAIV) is continuously circulating in many Asian countries and threatening poultry industry and human population. Vaccination is the best strategy to control H5N1 HPAIV infection in poultry and transmission to human population. The aim of this study is to identify new temperature-sensitive (ts) mutations for developing recombinant avian live attenuated H5N1 influenza vaccine.

****Findings**:**

A “6 + 2” recombinant virus C4/W1 that contained NA gene and modified HA gene from virus A/chicken/Hubei/327/2004 (H5N1) (C4), and six internal genes from virus A/duck/Hubei/W1/2004 (H9N2) (W1) was generated using reverse genetics and subsequently passaged in chicken eggs at progressively lower temperatures (32°C, 28°C and 25°C). The resulting virus acquired ts phenotype and one of its amino acid mutations, PA (F35S), was identified as ts mutation. Furthermore, when used as live attenuated vaccine, the recombinant virus with this ts mutation PA (F35S) provided efficient protection for chickens against H5N1 HPAIV infection.

**Conclusions:**

These findings highlight the potential of the new ts mutation PA (F35S) in developing recombinant avian live attenuated H5N1 influenza vaccine.

## Findings

H5N1 HPAIV is continuously circulating in many Asian countries and threatening poultry industry and human population. An effective vaccine to protect poultry from infections of H5N1 HPAIV is needed not only to reduce economic losses in the poultry industry, but also to minimize virus transmission from infected poultry to humans [1].

Live attenuated influenza vaccine (LAIV) with needle-free intranasal administration and cross-protection is attractive in controlling pandemic influenza. Recombinant influenza virus with C-terminal truncated NS1, deficient NA, cleavage-site modified HA or cytoplasmatic-tail deleted M2 has been reported as LAIV [2-5]. Besides these, low-temperature passage has been commonly used to acquire LAIV with ts phenotype [6-8]. The ts mutations were identified in internal genes of the LAIVs [9], and some LAIVs were subsequently used as master donor virus (MDV) to generate new LAIVs by reassortment with two surface glycoprotein genes from currently epidemic strains [10-13].

LAIV has been widely used in humans, while rare LAIV was reported to be used for poultry. Lee *et al.* developed an avian H9N2 LAIV by successively passaged a wild-type H9N2 influenza virus A/Chicken/Korea/S1/03 in embryonated chicken eggs at progressively lower temperatures [7]. Although the ts mutations of the avian H9N2 LAIV were not identified, some of the amino acid mutations (PB2^N265S^, PB1^K391E/E581G/A661T^ and NP^D34G^) were similar to the ts mutations of the MDV A/Ann Arbor/6/60 (H2N2). However, transferring these ts mutations (PB2^N265S^, PB1^K391E/E581G/A661T^ and NP^D34G^) into the corresponding internal genes of an avian H9N2 influenza virus A/Guinea Fowl/Hong Kong/WF10/99 could not sufficiently result in an avian live attenuated H9N2 influenza backbone, further introduction of an HA tag into PB1 gene was needed [14]. This strategy of generating avian live attenuated H9N2 influenza backbone seemed inconvenient, more simple ts mutations needed to be identified.

Therefore, the aim of this study was to acquire new and simple ts mutations within an avian H9N2 influenza backbone for developing recombinant avian live attenuated H5N1 influenza vaccine. All experiments with H5N1 HPAIV were performed in ABSL-3 containment facility. We initially generated a “6 + 2” recombinant virus C4/W1 that contained NA gene and modified HA gene (cleavage site was changed from PQRERRRKKR↓G to low pathogenic characteristic PQIETR↓G) [15] from H5N1 influenza virus A/Chicken/Hubei/327/2004 (C4) [16], and six internal genes from H9N2 influenza virus A/Duck/Hubei/W1/2004 (W1) [17]. Recombinant virus C4/W1 was then serially passaged in chicken eggs for 10 times at 32°C and 28°C respectively and 15 times at 25°C [7,8].

**Figure 1:**
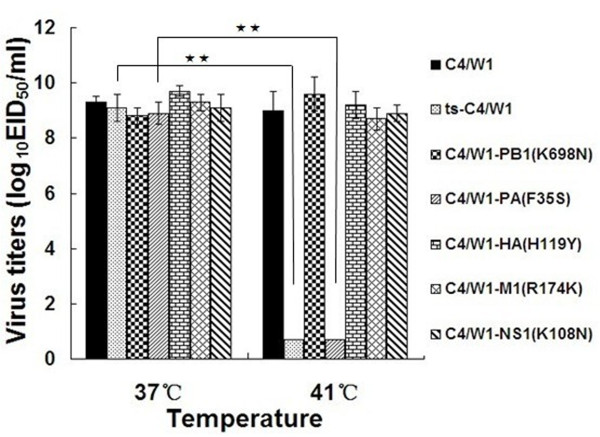
**Temperature-sensitive phenotype of the recombinant viruses.** Six 10-day-old embryonated SPF chicken eggs were inoculated with 10^3^ EID_50_ (0.2 ml/egg) of the recombinant viruses and incubated at 37°C and 41°C for 48 hours. Viral titers were determined by log_10_EID_50_/ml at 37°C. The detection limit is 0.7 log_10_EID_50_/ml. The statistical analysis was performed using Student’s *t* test (★★, *p* < 0.01; ★, *p* < 0.05).

The ts phenotype of this low-temperature passaged virus was evaluated. Six 10-day-old specific-pathogen-free (SPF) chicken eggs were inoculated with 10^3^ EID_50_ of the low-temperature passaged virus (0.2 ml/egg) and incubated at 37°C or 41°C for 48 hours, the parent virus C4/W1 was used as comparison. Allantoic fluids were collected and viral titers were determined by 50% egg infectious dose (EID_50_) at 37°C. Virus could be considered ts if it displayed 100-fold or greater reduction in viral titers at 41°C compared with that observed at 37°C. As shown in Figure 1, virus C4/W1 and the low-temperature passaged virus grew efficiently at 37°C (9.3 or 9.1 log_10_ EID_50_/ml, respectively). Nevertheless, at 41°C, the virus C4/W1 still grew up to high viral titers (9.0 log_10_ EID_50_/ml), while the low-temperature passaged virus was highly impaired in growth (under limited detection). These results indicated that the low-temperature passaged virus acquired ts phenotype and was named ts-C4/W1.

Subsequently, eight gene segments of the virus ts-C4/W1 were sequenced and compared with that of virus C4/W1. Five amino acid mutations were identified in the genome of the virus ts-C4/W1. They were PB1 (K698N), PA (F35S), HA (H119Y), M1 (R174K) and NS1 (K108N) (data not shown). To determine which amino acid mutation or mutations contributed to the ts phenotype of the virus ts-C4/W1, the five amino acid mutations were individually introduced into virus C4/W1 and five single-mutation recombinant viruses were generated. The ts phenotype of these single-mutation recombinant viruses was evaluated. As shown in Figure 1, four of the five single-mutation recombinant viruses including C4/W1-PB1(K698N), C4/W1-HA(H119Y), C4/W1-M1(R174K) and C4/W1-NS1(K108N) did not display ts phenotype, they grew up to similar titers at 37°C or 41°C. The rest single-mutation recombinant virus C4/W1-PA (F35S) grew efficiently at °C (8.9 log_10_ EID_50_/ml) in chicken eggs, but was highly impaired in growth at 41°C(under limited detection). These results indicated that PA (F35S) was the ts mutation.

To determine whether recombinant virus with the ts mutation PA (F35S) was attenuated in chickens, pathogenicity and growth property of the single-mutation recombinant virus C4/W1-PA (F35S) was assessed, viruses ts-C4/W1 and C4/W1 were used as comparison. Animal studies were performed according to protocols approved by the Hubei Provincial Animal Care and Use Committee. The intravenous pathogenicity index (IVPI) was performed [15] and indicated that virus C4/W1-PA (F35S) (IVPI = 0) as well as virus ts-C4/W1 (IVPI = 0) was more attenuated than virus C4/W1 (IVPI = 0.8). To assess growth property, groups of 3-week-old White Leghorn chickens were inoculated intranasally (i.n.) with 0.2 ml 10^6^ EID_50_ of the single-mutation recombinant virus C4/W1-PA (F35S), ts-C4/W1 or C4/W1, At 3 and 5 days post-inoculation (p.i.), four chickens in each group were sacrificed, tracheal swabs, cloacal swabs and lungs were collected in 1 ml phosphate buffered saline (PBS) containing penicillin and streptomycin, lungs were homogenized. Viral titers were determined by EID_50_ at 37°C. Virus C4/W1 grew efficiently in chickens and could be detected in tracheal swabs, cloacal swabs and lungs of inoculated chickens with high viral titers at 3 and 5 days p.i.; virus ts-C4/W1 couldn’t be detected in any swabs or lungs of the inoculated chickens; while virus C4/W1-PA (F35S) could only be detected in the tracheal swabs of inoculated chickens at 3 days p.i. with much less viral titers (1.8 log_10_EID_50_/ml) (Table 1). All these data showed that the single-mutation recombinant virus C4/W1-PA (F35S) as well as virus ts-C4/W1 was non-pathogenic to chickens and highly impaired in growth in chickens, which was critically important as LAIV.

**Table 1:** Pathogenicity and growth property of recombinant viruses in chickens and mice

**Viruses**	**IVPI**	**Virus replication in chickens**^**#**^**(log**_**10**_**EID**_**50**_**/ml ± SE)**						**MLD**_**50**_ **(log**_**10**_**EID**_**50**_ **/50 μl)**	**Virus replication in mice**^**&**^**(log**_**10**_**EID**_**50**_**/ml ± SE)**	
		**Day 3**			**Day 5**					
		**Trachea**	**Cloacal**	**Lungs**	**Trachea**	**Cloacal**	**Lungs**		**Day 3**	**Day 6**
C4/W1	0.8	4/4* (3.9 ± 0.4)	4/4 (2.4 ± 0.5)	4/4 (3.5 ± 0.2)	4/4 (5.4 ± 0.2)	3/4 (3.3 ± 0.3)	4/4 (4.7 ± 0.6)	6.5	3/3* (4.6 ± 0.2)	3/3 (3.3 ± 0.3)
ts-C4/W1	0	0/4	0/4	0/4	0/4	0/4	0/4	>8.0	2/3 (1.6 ± 0.1)	0/3
C4/W1-PA(F35S)	0	4/4 (1.8 ± 0.3)	0/4	0/4	0/4	0/4	0/4	>8.0	3/3 (3.4 ± 0.3)	2/3 (2.5 ± 0.1)

Note: IVPI, intravenous pathogenicity index; MLD_50_, 50% mouse lethal dose; EID_50_, 50% egg infectious dose; SE, standard errors.

^**#**^ Groups of chickens were inoculated intranasally with 0.2 ml 10^6^ EID_50_ of the indicated viruses. At 3 and 5 days post-inoculation, viral titers in tracheal swabs, cloacal swabs and lungs were determined by testing their EID_50_ at 37°C, values are the means ± standard errors.

^**&**^Groups of mice were inoculated intranasally with 50 μl 10^6^ EID_50_ of the indicated viruses. At 3 and 6 days post-inoculation, viral titers in lungs were determined by testing their EID_50_ at 37°C, values are the means ± standard errors.

^*^ NO. of chickens or mice with detectable virus/total chickens or mice used.

In addition, live attenuated vaccine must be non-pathogenic to vaccine operators before used in poultry. Here, mice were used to assess the virulence of virus C4/W1-PA (F35S). The fifty percent mouse lethal dose (MLD_50_) was determined as described previously [15]. As shown in Table 1, virus C4/W1-PA (F35S) and virus ts-C4/W1 were more attenuated (MLD_50_ > 8.0 log_10_EID_50_/50 μl) in mice than virus C4/W1 (MLD_50_ = 6.5 log_10_EID_50_/50 μl). This phenotype of attenuation could also be observed from body weight loss of mice infected i.n. with 50 μl 10^6^ EID_50_ of virus C4/W1-PA (F35S), ts-C4/W1 or C4/W1. Virus C4/W1 caused maximum 20% of initial body weight loss, while no apparent body weight loss was observed when mice infected with virus C4/W1-PA (F35S) or ts-C4/W1 (Data not shown). Their growth ability in mice was also determined. Groups of 6-week-old female BALB/c mice were inoculated i.n. with 50 μl 10^6^ EID_50_ of virus C4/W1-PA (F35S), ts-C4/W1 or C4/W1. At days 3 and 6 p.i., three mice in each group were sacrificed, and lungs were removed and homogenized in 1 ml PBS containing penicillin and streptomycin. Viral titers were determined by EID_50_ at 37°C. The results showed that viruses C4/W1-PA (F35S) and ts-C4/W1 grew up to lower titers than that of virus C4/W1 at 3 or 6 days p.i. (Table 1).

The PA subunit was reported to be critical in modulating ribonucleoprotein (RNP) activity under thermal stress (Kashiwagi *et al.*, 2010) [18]. Effects of the ts mutation PA (F35S) on polymerase activity was analyzed at different temperatures (37°C and 41°C) using minigenome reconstitution assay as described previously (Sun *et al.*, 2010) [19]. The RNP components of virus C4/W1 and C4/W1-PA(F35S) showed similar Luciferase activity at 37°C. However, at 41°C, the Luciferase activity of the RNP components of virus C4/W1 was 5.3-fold that of virus C4/W1-PA(F35S) (data not shown). The position 35 was near the endonuclease active site 41 of PA (Yuan *et al.*, 2009) [20], whether the amino acid mutation PA(F35S) restricted the endonuclease activity of PA to some extent under thermal stress needed to be identified.

To determine the protective efficacy of the indicated vaccines in chickens, groups of twelve 3-week-old White Leghorn chickens were inoculated i.n. with 0.2 ml 10^6^ EID_50_ of the virus C4/W1-PA (F35S), ts-C4/W1 or 0.2 ml PBS. Three weeks post-vaccination, six chickens in each group were challenged with 100 50% chicken lethal dose (CLD_50_) of either virus C4 (clade 9) or virus A/Duck/Hubei/hangmei01/2006 (H5N1) (D6) (clade 2.3.4) (Zou *et al.*, 2009) [21], tracheal and cloacal swabs were collected at days 3 post-challenge (p.c.) for virus titration and chickens were observed for death and disease signs for two weeks. As shown in Table 2, virus C4/W1-PA (F35S) induced detectable HI antibody titers and can provide efficient protection against the same clade or cross-clade H5N1 HPAIV with undetectable viral titers from tracheal or cloacal swabs at 3 days p.c., while all the chickens received with virus ts-C4/W1 or PBS died.

**Table 2:** Protective efficacy of the live attenuated H5N1 influenza vaccine in chickens

**Vaccine groups**^**#**^	**Challengingvirus**	**Positive/total No. (HI antibody, log**_**2**_**)before challenge**	**Death rate**	**Shedding/total (log**_**10**_**EID**_**50**_**/ml ± SE)**	
				**trachea**	**cloacal**
ts-C4/W1	C4	0/6	6/6^**$**^	ND (dead^**&**^)	ND (dead)
	D6	0/6	6/6	ND (dead)	ND (dead)
C4/W1-PA(F35S)	C4	6/6^*^ (2.3)	0/6	0/6	0/6
	D6	5/6 (2.6)	0/6	0/6	0/6
PBS	C4	0/6	6/6	ND (dead)	ND (dead)
	D6	0/6	6/6	ND (dead)	ND (dead)

Different sublineages of avian H9N2 influenza virus showed different pathogenicity to chickens or mice. Virus of A/Quail/Hong Kong/G1/97 sublineage with internal genes similar to the highly pathogenic Hong Kong/97 H5N1 influenza virus killed mice and spread to mouse brain, while virus of A/Duck/Hong Kong/Y280/97 H9N2 (Y280) sublineage were nonpathogenic to chickens or mice (Guo *et al.*, 2000) [22]. Choosing a nonpathogenic H9N2 influenza virus as backbone might be safer and the stain W1 used in this study as avian backbone was the Y280-sublineage.

In light of rare LAIV used for poultry, we highlighted the potential of the new and simple ts mutation PA (F35S) in developing recombinant avian H5N1 LAIV with avian H9N2 influenza virus as backbone.

## Competing interests

The authors declare that they have no competing interests.

## Authors’ contribution

WZ and JT performed the whole experiments. ZZ and HC analyzed the data. MJ designed the study and supervised the experiments. All authors read and approved the manuscript.
